# Traumatic Brain Injury in Different Age Groups

**DOI:** 10.3390/jcm11226739

**Published:** 2022-11-14

**Authors:** Abhijit Vijay Lele

**Affiliations:** Department of Anesthesiology, Neurocritical Care Service, Harborview Medical Center, The University of Washington, Seattle, WA 98104, USA; abhijit2@uw.edu; Tel.: +1-206-744-7598

Traumatic Brain Injury (TBI) is a global health burden. It is estimated that more than 69 million (95% confidence interval 64–74 million) individuals are estimated to suffer from TBI from all causes each year, with the Southeast Asian and Western Pacific regions experiencing the greatest overall burden of disease [1]. Admission patterns may differ by age [2], with the very young [3] and the elderly [4] at high risk for TBI. Falls are the most common mechanism of injury in 0–4 year olds [5] and the elderly [6].

Different age groups may not receive the same diagnostic testing and therapeutic interventions. Intracranial pressure monitoring may be infrequently used in children [7], despite the publication of pediatric Brain Trauma Foundation guidelines. The management intensity (defined as trauma team activation, advanced TBI imaging, invasive intracranial pressure monitoring, ventilator treatment, surgical evacuation of mass lesions, and decompressive craniectomy) of hospitalized patients with TBI may be decreased with advanced age, regardless of illness severity. Low management intensity may be associated with an increased risk of 30-day mortality [8]. Shimoda et al. has reported similar observations from a study using the Japan Neurotrauma Data Bank [9].

The outcomes of TBI vary with age [10]. In a study by Garza et. al, the >65 age group had a four-fold increased risk for unfavorable outcomes compared to the 18–34 group, this effect being most pronounced after mild TBI [2]. However, recent trends in the reduction in in-hospital mortality in moderate–severe TBI patients from the U.S may be encouraging [11]. TBI severity, pupillary reactivity, coagulation status, the need for blood product transfusion and acute bleeding, as well as basal cisterns obliteration found in head CT, may be associated with outcome of elderly patients with TBIs that needed surgical management [12].

Advancements in our understanding of the epidemiology and pathophysiology of TBI have resulted in evidence-based guidelines for pre-hospital [13] and intra-hospital care in adults [14] and children [15]. The evolution of the Brain Trauma Foundation’s TBI guidelines [16] and their correlation with published TBI research [17] highlight ongoing efforts to advance the quality of evidence for TBI care. Some of the advancements in TBI care include the Level I evidence against the use of corticosteroids to improve clinical outcomes, based on the results of the CRASH-1 trial [18]. The6 fourth edition of the Brain Trauma Foundation Guidelines has recommended blood pressure targets for different age groups (≥100 mm Hg for patients aged 50–69 years and ≥110 mm Hg for patients aged 15–59 or above 70 years) [14]. The recently published CRASH-3 trial indicates that tranexamic acid is safe in patients with TBI, and that treatment within three hours of injury reduces head injury-related death [19]. In patients with an intracranial pressure of more than 20 mm Hg after traumatic brain injury, therapeutic hypothermia plus standard care to reduce intracranial pressure did not result in outcomes better than those with standard care alone, based on results from the Eurotherm3235 trial [20]. The only randomized controlled trial related to intracranial pressure monitoring concluded that for patients with severe traumatic brain injury, care focused on maintaining monitored intracranial pressure at 20 mm Hg or less was not shown to be superior to care based on imaging and clinical examination [21].

We have advanced our understanding of the associations between adherence to trauma quality metrics and outcomes after TBI. The most common quality metric adhered to was the avoidance of steroids (Level I evidence) to improve TBI outcomes. Alali et al. demonstrated that hospitals with higher rates of ICP monitoring use were associated with lower mortality (adjusted odds ratio 0.52 (95% CI, 0.35–0.78)) in the quartile of hospitals with the highest use, compared to the lowest [22]. The use of end-tidal carbon dioxide in the emergency room is also a quality metric that targets the avoidance of inadvertent hypocarbia in patients at risk for cerebral hypoperfusion, especially those not being treated for an intracranial pressure crisis. A study by Thamjamrassri et al. suggests that two-thirds of children with complex mild TBI experience incomplete functional recovery at 1 year [23].

Despite several advances in TBI care, several knowledge gaps remain. Specifically, for elderly TBI patients, the confounding effects of pre-existing co-morbidities, frailty, and their impact on outcomes remain unknown [24]. The withdrawal of life-sustaining measures may occur early in as many as half of the patients aged 16 years and older, mostly in patients with severe TBI affecting brainstem reflexes [25]. Whether this trend implies self-fulfilling prophecies or an advancement in our understanding of long-term prognosis is unknown.

TBI outcomes may be impacted by available local resources, which may vary due to World Bank country income level. The use of intracranial pressure monitoring and management may vary across countries [26]. Sub-optimal pre-hospital care in low/middle-income countries prevails, and very little evidence exists in informing treatment in older patients (who comprise 30–40% of all TBI cases) [27]. The knowledge gained in adults with TBI may influence TBI care in children.

A number of TBI-related clinical trials (the majority being interventional) are actively recruiting adult and pediatric study participants [28]. These include more than 50 clinical trials enrolling children 0–17 years of age, more than 200 enrolling adults 18–65 years of age, and an equal number enrolling patients aged 65 years and older. These trials are being conducted in various regions around the world, namely, North America (*n* = 192), Africa (*n* = 6), East Asia (*n* = 17), Europe (*n* = 67), the Middle East (*n* = 5), North Asia (*n* = 4), Pacifica (*n* = 5), South America (*n* = 4), South Asia (*n* = 3), and Southeast Asia (*n* = 2).
